# Switching to Daily Doravirine/Islatravir (100/0.25 mg) Maintains Viral Suppression Through Week 48 Despite Baseline Non-Nucleoside Reverse Transcriptase Inhibitor Resistance–Associated Mutations or M184I/V in Proviral DNA

**DOI:** 10.1093/infdis/jiag257

**Published:** 2026-05-29

**Authors:** Tracy L Diamond, Anjana Grandhi, Monica Fuszard, Yayun Xu, Uchechukwu Nwoke, Ying Zhang, Stephanie O Klopfer, Karen Eves, Jaime Benner, Mengchun Li, Wayne Greaves, Rima Lahoulou, Jason Kim, Michelle C Fox, Ernest Asante-Appiah

**Affiliations:** MRL, Merck & Co., Inc., Rahway, New Jersey, USA; MRL, Merck & Co., Inc., Rahway, New Jersey, USA; MRL, Merck & Co., Inc., Rahway, New Jersey, USA; MRL, Merck & Co., Inc., Rahway, New Jersey, USA; MRL, Merck & Co., Inc., Rahway, New Jersey, USA; MRL, Merck & Co., Inc., Rahway, New Jersey, USA; MRL, Merck & Co., Inc., Rahway, New Jersey, USA; MRL, Merck & Co., Inc., Rahway, New Jersey, USA; MRL, Merck & Co., Inc., Rahway, New Jersey, USA; MRL, Merck & Co., Inc., Rahway, New Jersey, USA; MRL, Merck & Co., Inc., Rahway, New Jersey, USA; MRL, Merck & Co., Inc., Rahway, New Jersey, USA; MRL, Merck & Co., Inc., Rahway, New Jersey, USA; MRL, Merck & Co., Inc., Rahway, New Jersey, USA; MRL, Merck & Co., Inc., Rahway, New Jersey, USA

**Keywords:** HIV-1, treatment, virologically suppressed, resistance analysis, oral

## Abstract

**Background:**

Doravirine/islatravir (DOR/ISL) is a 2-drug, single-tablet regimen in clinical development for once-daily treatment of adults living with HIV-1. We examined the impact of baseline resistance-associated mutations (RAMs) in proviral DNA on the response to DOR/ISL (100/0.25 mg) in virologically suppressed participants in three phase 3 studies.

**Methods:**

Samples were collected pre-dose from participants in studies P051 (NCT05631093), P052 (NCT05630755), and P054 (NCT05766501) to evaluate RAMs in proviral DNA at baseline. The prevalence of baseline non-nucleoside reverse transcriptase inhibitor (NNRTI) RAMs and M184I/V, and their impact on virologic suppression (HIV-1 RNA <50 copies/mL) at week 48, as well as on types of viremia (confirmed HIV-1 RNA ≥200 copies/mL, low-level viremia, and transient viremia), was examined. Samples from participants with confirmed HIV-1 RNA ≥200 copies/mL or who discontinued from treatment with HIV-1 RNA ≥200 copies/mL were assessed for viral drug resistance.

**Results:**

Among 1227 participants who switched to DOR/ISL, 323 (26.3%) had baseline NNRTI RAMs and 70 (5.7%) had baseline M184I/V. At week 48, DOR/ISL maintained HIV-1 RNA <50 copies/mL in ≥88% of participants with and/or without NNRTI RAMs and/or M184I/V detected in proviral DNA at baseline. The percentage of participants in each viremia category was comparable with regard to the presence or absence of NNRTI RAMs and/or M184I/V. No treatment-emergent resistance to DOR or ISL was observed in the 7 participants who met criteria for postbaseline resistance testing.

**Conclusions:**

Baseline NNRTI RAMs and M184I/V in proviral DNA did not impact virologic outcomes through 48 weeks after switching to DOR/ISL (100/0.25 mg).

**Clinical Trials Registration:**

ClinicalTrials.gov: NCT05631093, NCT05630755, NCT05766501.

## Teaser

Doravirine/islatravir (DOR/ISL) is an oral regimen in development for once-daily treatment of HIV-1. In virologically suppressed participants, baseline non-nucleoside reverse transcriptase inhibitor resistance–associated mutations and M184I/V in proviral DNA did not affect virologic outcomes through 48 weeks after switching to DOR/ISL (100/0.25 mg).

Since the introduction of antiretrovirals in the 1980s, HIV-1 (HIV) has shown a persistent capability of developing resistance to antiretroviral therapy (ART) [1, 2]. This understanding led to combination regimens and the need to identify therapies with higher barriers to resistance [1, 2]. Maintaining good adherence remains essential for preventing virologic failure and drug resistance, and the use of fixed-dose combinations and once-daily formulations helps reduce pill burden and potentially improves adherence [3, 4].

Doravirine/islatravir (DOR/ISL), a 2-drug, single-tablet regimen comprising DOR, a non-nucleoside reverse transcriptase inhibitor (NNRTI), and ISL, a nucleoside reverse transcriptase translocation inhibitor. DOR/ISL is approved in the US and Japan for once-daily treatment of virologically suppressed people living with HIV. DOR/ISL (100/0.25 mg) is being evaluated in 3 ongoing phase 3 studies: MK-8591A-051 (P051; NCT05631093), MK-8591A-052 (P052; NCT05630755), and MK-8591A-054 (P054; NCT05766501) in virologically suppressed adults.

DOR exhibits potent in vitro activity against wild-type HIV and variants bearing common NNRTI resistance–associated mutations (RAMs), including K103N, E138K, Y181C, and G190A [5]. The following RAMs in reverse transcriptase (RT) have been associated with resistance to DOR in clinical studies or have demonstrated reduced susceptibility to DOR in vitro: V106A/M, V108I, Y188L, H221Y, P225H, F227C/L/V, M230I/L, L234I, P236I, or Y318F and were exclusionary in DOR/ISL phase 3 studies [6–11].

The resistance profile of ISL has been studied extensively in vitro through both resistance selection experiments and by evaluation of the potency of ISL against variants generated through site-directed mutagenesis or from clinical isolates bearing RAMs [12–16]. Only variants containing M184I or M184V (M184I/V) have been reproducibly shown to decrease the potency of ISL >4-fold. ISL 0.25 mg daily is predicted to achieve trough intracellular triphosphate (the active form of ISL) concentrations approximately 100-fold above the IC_50_ (9.74 ± 4.06 fmol per 10^6^ cells) [17, 18]. These levels result in inhibitory quotients 20- and 4-fold above the targets for efficacy against wild-type HIV and M184I/V variants, respectively. This primary resistance analysis from three phase 3 studies at week (W) 48 examined the prevalence of baseline RAMs in proviral DNA and their impact on the virologic response to DOR/ISL (100/0.25 mg) through W48. The emergence of RAMs in participants who met the criteria for postbaseline resistance testing (confirmed HIV RNA ≥200 copies/mL or discontinued with ≥200 copies/mL) was examined.

## METHODS

### Design of Clinical Studies

P051 is a randomized, active-controlled, open-label phase 3 study evaluating a switch to once-daily oral DOR/ISL (100/0.25 mg) [10]. Participants were aged ≥18 years with HIV RNA <50 copies/mL at screening and virologically suppressed for ≥3 consecutive months on a stable oral 2- or 3-drug (±pharmacokinetic [PK] booster) ART regimen. Overall, 553 participants were randomized (2:1), of whom 551 received ≥1 dose of study intervention: 366 participants switched from bART to DOR/ISL, 185 participants continued bART. This analysis reflects data collected through W48.

P052 is a randomized, active-controlled, double-blind phase 3 study evaluating a switch from once-daily bictegravir/emtricitabine/tenofovir alafenamide (BIC/FTC/TAF) to DOR/ISL (100/0.25 mg) in participants aged ≥18 years with HIV RNA <50 copies/mL at screening and virologically suppressed for ≥3 consecutive months on BIC/FTC/TAF [11]. A total of 514 participants were randomized 2:1 and 513 participants received ≥1 dose of study intervention: 342 participants switched from BIC/FTC/TAF to DOR/ISL, 171 participants continued BIC/FTC/TAF. This analysis reflects data collected through W48.

Participants in P051 and P052 were enrolled based on no reported history of prior virologic treatment failure on any past or current regimen and no documented resistance to DOR (based on the following RAMs in RT: V106A/M, V108I, Y188L, H221Y, P225H, F227C/L/V, M230I/L, L234I, P236L, or Y318F). Resistance testing was not a requirement for enrollment.

Open-label study P054 enrolled participants aged ≥18 years living with HIV who received once-daily DOR/ISL (100/0.75 mg) in previous phase 3 studies and were considered by the investigator to have derived clinical benefit from receiving DOR/ISL (100/0.75 mg) and for whom further treatment with DOR/ISL (100/0.25 mg) was considered clinically appropriate. Previous studies had similar inclusion/exclusion criteria, with no reported history of prior virologic treatment or resistance to DOR. All participants in P054 were treated with open-label DOR/ISL (100/0.25 mg). Written informed consent was obtained from all study participants before screening.

### Evaluation of Resistance From Clinical Samples

Whole blood samples collected at baseline (day 1, pre-dose) were assessed for RAMs in proviral DNA using the GenoSure Archive^®^ assay (Monogram Biosciences, a subsidiary of LabCorp, South San Francisco, CA, United States) [19]. If P054 results were unavailable due to assay failure, baseline samples from previous DOR/ISL (100/0.75 mg) studies were used instead. GenoSure Archive^®^ uses next-generation sequencing with a proprietary filter to exclude hypermutated reads. A reporting threshold of 10% was employed to minimize the impact of apolipoprotein B mRNA editing catalytic polypeptide-like (APOBEC)-induced hypermutation false positivity. Additionally, manual quality control was employed to examine sites of APOBEC-induced hypermutation. A reporting threshold of 3% was used for genomic positions unlikely to undergo APOBEC-induced changes.

Participants with confirmed HIV RNA ≥200 copies/mL or who discontinued with HIV RNA ≥200 copies/mL at any time met the criteria for postbaseline resistance analysis. Plasma samples collected at the time of viremia were assessed for development of drug resistance in viral RNA. Genotypic and phenotypic resistance assessments were performed using GenoSure PRIme^®^, PhenoSense^®^ Integrase, or PhenoSense^®^ assays (Monogram Biosciences), which are validated for testing samples with HIV viral loads ≥500 copies/mL. TheGenoSure PRIme^®^ assay uses next-generation sequencing with a reporting threshold of 10% to identify mutations compared with the reference strain (HIV clone NL43I151V; GenBank ID: KM390026.1). PhenoSense^®^ assays provide a quantitative measurement of antiretroviral activity against clinical isolates compared with reference virus [20].

### Resistance-Associated Mutations Evaluated for Each Protein and Drug Class

Resistance analysis evaluated RAMs associated with each viral protein (RT, integrase, and protease) and drug class based on the International AIDS Society-USA drug resistance mutations list and Stanford University HIV Drug Resistance Database (Table 1) [21, 22]. The list of RAMs includes major RAMs that confer larger reductions in susceptibility and can independently confer resistance and minor RAMs that occur after the emergence of a major RAM, confer smaller reductions in susceptibility, or may be accessory mutations that increase viral fitness. The list of DOR RAMs is based on in vitro resistance profiling, phenotypic resistance from clinical studies, and data from published literature [5–9]. As the antiretroviral regimens differed across the studies, each analysis focused on RAMs relevant to the ART used in that study (P051: RAMs in RT [NNRTI and NRTI], integrase [InSTI], and protease [PI]; P052: RAMs in RT [NNRTI and NRTI] and integrase [InSTI]; and P054: RAMs in RT [NNRTI and NRTI]).

**Table 1.** List of RAMs Evaluated for Each Target Protein and Inhibitor Class

Protein	ARV Class	Major RAMs	Minor RAMs
RT	NNRTI	L100I, K101E/P, K103N/S, V106A/M, V108I, E138A/G/K/Q/R, Y181C/I/V, Y188C/H/L, G190A/S, F227C/I/L/R/V, M230I/L, Y318F	V90I, A98G, K101H, K103R, V106I/T, V179D/F/L/T, Y188A/E/S, G190E, H221Y, P225H, F227H, L234I, P236L
	DOR-specific	V106A/M, Y188L, F227C/L/V, M230I/L, L234I, Y318F	V108I, H221Y, P225H, P236L
	NRTI	M41L, A62V, K65E/N/R, D67N, 69ins, K70E/R, L74V, V75I, F77L, Y115F, F116Y, Q151M, M184I/V, L210W, T215F/Y, K219E/Q	E44D, T69D/N, V118I, T215A/C/D/E/G/H/I/L/N/S/V, K219N/R
Integrase	InSTI	T66I, E92Q, G118R, F121Y, G140R, Y143C/H/R, S147G, Q148H/K/R, S153A, N155H/S, R263K	M50I, H51Y, T66A/K, L74M, E92G, T97A, E138A/K/T, G140A/C/S, S153F/Y, E157K/Q, G163K/R
Protease	PI	D30N, V32I, M46I/L, I47A/V, G48V, I50L/V, I54L/M/V, Q58E, G73A/C/S/T, T74P, L76V, V82A/F/L/S/T, N83D, I84V, N88S, L90M	L10F/I/R/V, V11I, K20M/R/T, L24I, L33F, M36I/L/V, K43T, F53Y, I54A/S/T, I62V, H69K/R, A71T/V, V77I, V82M, I84A/C, I85V, N88D, L89I/M/V

Abbreviations: ARV, antiretroviral; DOR, doravirine; InSTI, integrase strand transfer inhibitor; NNRTI, non-nucleoside reverse transcriptase inhibitor; NRTI, nucleos(t)ide reverse transcriptase inhibitor; PI, protease inhibitor; RAM, resistance-associated mutation; RT, reverse transcriptase.

Baseline DOR resistance was assessed against all detected NNRTI RAMs, and ISL resistance was assessed against M184I/V, which remains the only RAM associated with a reduction in ISL potency. For postbaseline analysis in participants with viremia, analysis included any missense mutations at a codon associated with resistance in RT.

### Analysis of Viremia

Plasma viremia was monitored, per protocol, throughout each study using the Cobas^®^ HIV assay (Roche Diagnostics, Basel, Switzerland). For samples with HIV RNA ≥50 copies/mL, reflex testing was performed on samples obtained at the same visit. The management of participants was guided by the reflex HIV RNA results when available. Participants with virologic data within the W48 window were evaluated based upon HIV RNA ≥50 copies/mL or <50 copies/mL (viral suppression). Participants who discontinued study intervention prior to W48 due to lack of efficacy or other reasons, and whose last on-treatment HIV RNA results were ≥50 copies/mL, were categorized as having HIV RNA ≥50 copies/mL at W48.

Participants were further assigned to one of the following mutually exclusive categories based on their viremia profiles over the 48-week period: discontinued with 2 consecutive HIV RNA measurements ≥200 copies/mL; low-level viremia, defined as confirmed HIV RNA ≥50 copies/mL that did not meet the criteria for discontinuation; and transient viremia (viral blips), defined as a temporary on-treatment increase in HIV RNA ≥50 copies/mL preceded and followed by HIV RNA values <50 copies/mL.

### Pharmacokinetics Plasma Concentrations of Doravirine and Islatravir

Adherence was evaluated by plasma concentration assessment of DOR and/or ISL by liquid chromatography–tandem mass spectrometry (LC-MS/MS; Syneos Health, Québec City, QC, Canada).

## RESULTS

### Baseline Proviral DNA Resistance Analysis

Proviral DNA resistance data were available for 90.5% of participants (1545/1708) across the studies: 88.2% from P051 (group 1: DOR/ISL, 322/368 [87.5%]; group 2: bART, 166/185 [89.7%]), 89.7% from P052 (group 1: DOR/ISL, 309/343 [90.1%]; group 2: BIC/FTC/TAF, 152/171 [88.9%]), and 93.0% from P054 (596/641). Among P054 participants with available proviral DNA, the baseline sample was used for 93.0% of participants; the remaining 7.0% of baseline data were derived from samples obtained during previous phase 3 studies of DOR/ISL (100/0.75 mg), as P054 baseline samples were not available.

The prevalence of RAMs in RT, protease, and integrase that could potentially affect susceptibility to DOR/ISL or bART regimens was evaluated. A summary of baseline RAMs detected at >5% in proviral DNA is provided (Table 2). Detailed prevalence data for all baseline RAMs and combinations thereof are included in Supplementary Tables 1–3. The prevalence of class-specific RAMs was comparable between studies and treatment groups. Among 1227 participants who switched to DOR/ISL (100/0.25 mg) across P051, P052, and P054, 323 (26.3%) had baseline NNRTI RAMs and 70 (5.7%) had baseline M184I/V (90% of which had M184V observed). Most of the RAMs observed in >5% of participants were minor RAMs. K103N/R/S, E138K/Q, and M184I/V were the most prevalent RAMs observed in RT at baseline.

**Table 2.** Prevalence of RAMs at Baseline in Proviral DNA Observed in >5% of Participants in Any Study Arm

	P051		P052		P054	Pooled
	DOR/ISL (100 mg/0.25 mg)	bART	DOR/ISL (100 mg/0.25 mg)	BIC/FTC/TAF	DOR/ISL (100 mg/0.25 mg)	DOR/ISL (100 mg/0.25 mg)
	N = 322	N = 166	N = 309	N = 152	N = 596	N = 1227
	n (%)	n (%)	n (%)	n (%)	n (%)	n (%)
**RT**						
** Any NNRTI RAM**	**79** (**24.5)**	**33** (**19.9)**	**82** (**26.5)**	**41** (**27.0)**	**162** (**27.2)**	**323** (**26.3)**
K103N/R/S	34 (10.6)	11 (6.6)	38 (12.3)	16 (10.5)	53 (8.9)	125 (10.2)
E138A/G/K/Q/R	21 (6.5)	7 (4.2)	18 (5.8)	9 (5.9)	39 (6.5)	78 (6.4)
						
** Any NRTI RAM**	**59** (**18.3)**	**25** (**15.1)**	**62** (**20.1)**	**32** (**21.1)**	**132** (**22.1)**	**253** (**20.6)**
V118I	20 (6.2)	11 (6.6)	18 (5.8)	10 (6.6)	49 (8.2)	87 (7.1)
M184I/V	20 (6.2)	5 (3.0)	24 (7.8)	10 (6.6)	26 (4.4)	70 (5.7)
**Integrase**						
** Any InSTI RAM**	**124** (**38.5)**	**68** (**41.0)**	**88** (**28.5)**	**37** (**24.3)**		
M50I	91 (28.3)	53 (31.9)	49 (15.9)	23 (15.1)		
E157K/Q	13 (4.0)	9 (5.4)	19 (6.1)	9 (5.9)		
**Protease**						
** Any PI RAM**	**280** (**87.0)**	**149** (**89.8)**				
M36I/L/V	166 (51.6)	87 (52.4)				
V77I	86 (26.7)	44 (26.5)				
I62V	89 (27.6)	45 (27.1)				
H69K/R	123 (38.2)	72 (43.4)				
L89I/M/V	109 (33.9)	68 (41.0)				
A71T/V	43 (13.4)	18 (10.8)				
L10F/I/R/V	42 (13.0)	25 (15.1)				
K20M/R/T	48 (14.9)	22 (13.3)				

RAMs were sometimes present as mixtures with wild-type or other mutations within the same codon.

Abbreviations: bART, baseline antiretroviral therapy; BIC, bictegravir; DOR, doravirine; FTC, emtricitabine; InSTI, integrase strand transfer inhibitor; ISL, islatravir; N, number of participants with proviral DNA data at baseline; n, participants in the category; NNRTI, non-nucleoside reverse transcriptase inhibitor; NRTI, nucleos(t)ide reverse transcriptase inhibitor; PI, protease inhibitor; RAM, resistance-associated mutation; RT, reverse transcriptase; TAF, tenofovir alafenamide.

Although previously documented resistance to DOR was exclusionary, DOR RAMs were detected in 3.5% of participants (43/1227) that switched to DOR/ISL (100/0.25 mg) across P051, P052, and P054 (Table 3). V108I was the most prevalent DOR RAM, observed in 1.0% of participants across the pooled DOR/ISL treatment groups.

**Table 3.** Prevalence of DOR RAMs at Baseline in Proviral DNA

	P051		P052		P054	Pooled
	DOR/ISL (100 mg/0.25 mg)	bART	DOR/ISL (100 mg/0.25 mg)	BIC/FTC/TAF	DOR/ISL (100 mg/0.25 mg)	DOR/ISL (100 mg/0.25 mg)
	N = 322	N = 166	N = 309	N = 152	N = 596	N = 1227
	n (%)	n (%)	n (%)	n (%)	n (%)	n (%)
**Any DOR RAM**	**7** (**2.2)**	**7** (**4.2)**	**15** (**4.9)**	**3** (**2.0)**	**21** (**3.5)**	**43** (**3.5)**
V108I	2 (0.6)	3 (1.8)	3 (1.0)	2 (1.3)	7 (1.2)	12 (1.0)
V106A/M	4 (1.2)	0	1 (0.3)	0	4 (0.7)	9 (0.7)
P225H	1 (0.3)	0	3 (1.0)	1 (0.7)	3 (0.5)	7 (0.6)
H221Y	0	1 (0.6)	4 (1.3)	1 (0.7)	3 (0.5)	7 (0.6)
M230I/L	0	0	2 (0.6)	0	2 (0.3)	4 (0.3)
F227C/L/V	1 (0.3)	1 (0.6)	0	0	2 (0.3)	3 (0.2)
Y188L	2 (0.6)	0	0	1 (0.7)	0	2 (0.2)
Y318F	0	1 (0.6)	2 (0.6)	0	0	2 (0.2)
L234I	0	0	1 (0.3)	0	0	1 (0.0)
P236L	0	1 (0.6)	0	0	0	0

RAMs were sometimes present as mixtures with wild-type or other mutations within the same codon.

Abbreviations: bART, baseline antiretroviral therapy; BIC, bictegravir; DOR, doravirine; FTC, emtricitabine; ISL, islatravir; N, number of participants with proviral DNA data at baseline; n, participants in the category; RAM, resistance-associated mutation; TAF, tenofovir alafenamide.

### Virologic Outcomes in Participants With Baseline Proviral DNA Resistance Data

Most participants with baseline proviral DNA data in each study and group (>92%) had virologic data available at W48. The percentage of participants with HIV <50 and ≥50 copies/mL was comparable across study and group (Table 4). Across the 3 studies, 98.4% of DOR/ISL participants with proviral DNA resistance data (1149/1227) maintained HIV RNA <50 copies/mL at W48. Comparable percentages of viral suppression at W48 were observed in participants with (87.9%–96.7%) or without (98.9%) baseline NNRTI RAMs and/or M184I/V.

**Table 4.** Virologic Outcomes at W48 in Participants With NNRTI RAMs or M184I/V at Baseline in Proviral DNA

Participant(s) With	P051		P052		P054	Pooled
	DOR/ISL (100 mg/0.25 mg)	bART	DOR/ISL (100 mg/0.25 mg)	BIC/FTC/TAF	DOR/ISL (100 mg/0.25 mg)	DOR/ISL (100 mg/0.25 mg)
**Baseline proviral DNA data**	**N = 322**	**N = 166**	**N = 309**	**N = 152**	**N = 596**	**N = 1227**
Virologic data at W48, n/N (%)	313/322 (97.2)	161/166 (97.0)	285/309 (92.2)	145/152 (95.4)	570/596 (95.6)	1168/1227 (95.2)
HIV RNA <50 c/mL at W48, n (%)	309 (98.7)	153 (95.0)	280 (98.2)	144 (99.3)	560 (98.2)	1149 (98.4)
HIV RNA ≥50 c/mL at W48, n (%)	4^a^ (1.3)	8 (5.0)	5 (1.8)	1 (0.7)	10 (1.8)	19^a^ (1.5)
**Baseline proviral DNA data and virologic data at W48**	**N = 313**	**N = 161**	**N = 285**	**N = 145**	**N = 570**	**N = 1168**
No NNRTI RAMs or M184I/V at baseline, n/N (%)	223/313 (71.2)	125/161 (77.6)	203/285 (71.2)	100/145 (69.0)	402/570 (70.5)	828/1168 (70.9)
HIV RNA <50 c/mL at W48, n (%)	222 (99.6)	119 (95.2)	200 (98.5)	99 (99.0)	397 (98.8)	819 (98.9)
HIV RNA ≥50 c/mL at W48, n (%)	1 (0.4)	6 (4.8)	3 (1.5)	1 (1.0)	5 (1.2)	9 (1.1)
Any NNRTI RAMs at baseline, n/N (%)	77/313 (24.6)	33/161 (20.5)	75/285 (26.3)	39/145 (26.9)	155/570 (27.2)	307/1168 (26.3)
HIV RNA <50 c/mL at W48, n (%)	74 (96.1)	31 (93.9)	73 (97.3)	39 (100.0)	150 (96.8)	297 (96.7)
HIV RNA ≥50 c/mL at W48, n (%)	3^a^ (3.9)	2 (6.1)	2 (2.7)	0	5 (3.2)	10^a^ (3.3)
M184I/V at baseline, n/N (%)	20/313 (6.4)	4/161 (2.5)	20/285 (7.0)	8/145 (5.5)	26/570 (4.6)	66/1168 (5.7)
HIV RNA <50 c/mL at W48, n (%)	19 (95.0)	4 (100.0)	18 (90.0)	8 (100.0)	25 (96.2)	62 (93.9)
HIV RNA ≥50 c/mL at W48, n (%)	1^a^ (5.0)	0	2 (10.0)	0	1 (3.8)	4^a^ (6.1)
Both NNRTI RAMs and M184I/V at baseline, n/N (%)	7/313 (2.2)	1/161 (0.6)	13/285 (3.5)	2/145 (1.4)	13/570 (2.3)	33/1168 (2.8)
HIV RNA <50 c/mL at W48, n (%)	6 (85.7)	1 (100.0)	11 (84.6)	2 (100.0)	12 (92.3)	29 (87.9)
HIV RNA ≥50 c/mL at W48, n (%)	1^a^ (14.3)	0	2 (15.4)	0	1 (7.7)	4^a^ (12.1)

Abbreviations: bART, baseline antiretroviral therapy; BIC, bictegravir; c, copies; DOR, doravirine; FTC, emtricitabine; ISL, islatravir; N, number of participants with proviral DNA data at baseline; n, participants in the category; NNRTI, non-nucleoside reverse transcriptase inhibitor; RAM, resistance-associated mutation; TAF, tenofovir alafenamide; W, week.

^a^Two participants that discontinued with confirmed HIV RNA ≥200 copies/mL in P051 were later determined to be ineligible for enrollment due to either a previous history of virologic failure (n = 1) or a previous documentation of DOR resistance (n = 1). Both had baseline NNRTI RAMs (including Y188L) and 1 had a baseline M184I/V RAM.

Additional analysis assessed the impact of baseline resistance in proviral DNA on viremia outcomes throughout the 48-week treatment period. This analysis assessed nonpredefined exploratory endpoints, including the number and percentage of participants who had transient viremia, low-level viremia, or discontinued with confirmed HIV RNA ≥200 copies/mL through W48 in each of the studies. The percentage of participants in each viremia category was comparable across each study/group (Table 5). The number of participants who discontinued due to viremia was low (0.4%–2.9%), and transient viremia was the most common category (5.5%–8.6%). The percentage of participants in each viremia category was comparable with regards to the presence or absence of NNRTI RAMs and/or M184I/V. Viremia outcomes were assessed in 6 DOR/ISL participants with M184I (n = 1) or M184V (n = 5) that were not mixed with wild-type M184; only 1 had any viremia (transient viremia) through W48.

**Table 5.** Viremia Outcomes Through W48 in Participants With Proviral DNA Data at Baseline

Participant(s) With		P051		P052		P054	Pooled
		DOR/ISL (100 mg/0.25 mg)	bART	DOR/ISL (100 mg/0.25 mg)	BIC/FTC/TAF	DOR/ISL (100 mg/0.25 mg)	DOR/ISL (100 mg/0.25 mg)
**Baseline proviral DNA data**	**All**	**N = 322**	**N = 166**	**N = 309**	**N = 152**	**N = 596**	**N = 1227**
	DISC (%)	3^a^ (0.9)	0	2 (0.6)	0	1 (0.2)	6^a^ (0.5)
	LLV (%)	5 (1.6)	6 (3.6)	5 (1.6)	2 (1.3)	13 (2.2)	23 (1.9)
	TV (%)	15 (4.7)	17 (10.2)	18 (5.8)	8 (5.3)	38 (6.4)	71 (5.8)
**No NNRTI RAMs or M184I/V**	**All**	**223**	**128**	**203**	**101**	**408**	**834**
	DISC (%)	0	0	2 (1.0)	0	1 (0.2)	3 (0.4)
	LLV (%)	4 (1.8)	6 (5.7)	0	2 (2.0)	3 (0.7)	7 (0.8)
	TV (%)	12 (5.4)	15 (9.0)	13 (6.4)	5 (5.0)	21 (5.1)	46 (5.5)
**NNRTI RAMs**	**All**	**79**	**33**	**82**	**41**	**162**	**323**
	DISC (%)	2^a^ (2.5)	0	0	0	0	2^a^ (0.6)
	LLV (%)	1 (1.3)	0	3 (3.7)	0	9 (5.6)	13 (4.0)
	TV (%)	3 (3.8)	2 (6.1)	4 (4.9)	2 (4.9)	14 (8.6)	21 (6.5)
**M184I/V**	**All**	**20**	**5**	**24**	**10**	**26**	**70**
	DISC (%)	1^a^ (5.0)	0	0	0	0	1^a^ (1.4)
	LLV (%)	0	0	2 (8.3)	0	1 (3.8)	3 (4.3)
	TV (%)	0	0	1 (4.2)	1 (10.0)	3 (11.5)	4 (5.7)
**NNRTI RAMs and M184I/V**	**All**	**7**	**1**	**15**	**4**	**13**	**35**
	DISC (%)	1^a^ (14.3)	0	0	0	0	1^a^ (2.9)
	LLV (%)	0	0	2 (13.3)	0	1 (3.8)	3 (8.6)
	TV (%)	0	0	1 (6.7)	0	2 (15.4)	3 (8.6)

Abbreviations: bART, baseline antiretroviral therapy; BIC, bictegravir; DISC, discontinued with confirmed HIV RNA ≥200 copies/mL; DOR, doravirine; FTC, emtricitabine; ISL, islatravir; LLV, low-level viremia; N, number of participants with proviral DNA data at baseline; NNRTI, non-nucleoside reverse transcriptase inhibitor; RAM, resistance-associated mutation; TAF, tenofovir alafenamide; TV, transient viremia; W, week.

^a^Includes participant(s) who were ineligible for enrollment due to either a previous history of virologic failure or a previous documentation of DOR resistance (Y188L).

Among DOR/ISL participants across the 3 studies, the number of individuals with both NNRTI RAMs and M184I/V at baseline was small (2.7%, 35/1277), and 7 of these participants had viremia (Table 5). Of the 7 participants with viremia, 1 discontinued with confirmed HIV RNA ≥200 copies/mL prior to W48 and was later determined to be ineligible for enrollment due to previous documentation of Y188L; 3 had low-level viremia (including 1 [P052] who resolved by W48, 1 participant (P052) who had treatment-emergent resistance to DOR based on a post hoc analysis [23] and withdrew from the study with low-level viremia prior to W48, and 1 (P054) who still had low-level viremia at W48); and 3 had single episodes of transient viremia ≥50 copies/mL through W48.

Among the 43 DOR/ISL participants across the 3 studies with DOR RAMs at baseline, 6 experienced viremia through W48. Of the 6 participants, 2 (both from P051) discontinued with confirmed HIV RNA ≥200 copies/mL; both were later determined to be ineligible for enrollment due to either a previous history of virologic failure (n = 1) or documented DOR resistance (Y188L; n = 1). The remaining 4 participants (all from P054) had single episodes of transient viremia through W48 (Table 6). Eight of the 43 DOR/ISL participants had both DOR RAMs and M184I/V across the 3 studies; 2 of these individuals experienced viremia (1 discontinued the study and another had transient viremia) through W48.

**Table 6.** Viremia Outcomes Through W48 in DOR/ISL Participants With DOR RAMs at Baseline

Participant(s) With		P051	P052	P054	Pooled
		DOR/ISL (100 mg/0.25 mg)	DOR/ISL (100 mg/0.25 mg)	DOR/ISL (100 mg/0.25 mg)	DOR/ISL (100 mg/0.25 mg)
**DOR RAMs**	**All**	7	15	21	**43**
	DISC (%)	2^a^ (28.6)	0	0	2^a^ (4.6)
	LLV (%)	0	0	0	0
	TV (%)	0	0	4 (19.0)	4 (9.3)
**DOR RAMs and M184I/V**	**All**	2	3	3	**8**
	DISC (%)	1^a^ (50.0)	0	0	1^a^ (12.5)
	LLV (%)	0	0	0	0
	TV (%)	0	0	1 (33.3)	1 (12.5)

Abbreviations: DISC, discontinued with confirmed HIV RNA ≥200 copies/mL; DOR, doravirine; ISL, islatravir; LLV, low-level viremia; RAM, resistance-associated mutation; TV, transient viremia; W, week.

^a^Includes participant(s) who were ineligible for enrollment due to either a previous history of virologic failure or a previous documentation of DOR resistance (Y188L).

### Postbaseline Resistance Analysis

Seven DOR/ISL participants (P051, n = 3; P052, n = 2; P054, n = 2) met the criteria for postbaseline resistance testing through W48, and their samples were submitted for genotypic and phenotypic analysis of viral RNA (Table 7). No treatment-emergent resistance to either DOR or ISL was observed through W48.

**Table 7.** Resistance Analysis in DOR/ISL Participants Who Discontinued With Confirmed HIV RNA ≥200 Copies/mL

Participant	Study	HIV Subtype	Baseline RAMs in Proviral DNA^a^	HIV RNA (copies/mL) for Sample (Study Week)	RAMs n Viral RNA	Treatment-Emergent RAMs	Phenotypic Fold-Shift
**Participants without postbaseline results due to assay failure**							
1	P052	B	NNRTI: none NRTI: none	287/486 (W36)^b^	Assay failed	N/A	Assay failed
2	P054	AE	NNRTI: V179V/I NRTI: none	332 (W12)	Assay failed	N/A	Assay failed
3^c^	P054	Assay failed	Assay failed	227 (W48)	Assay failed	N/A	Assay failed
**Participants who were later determined to be ineligible for enrollment^d^**							
4	P051	B	NNRTI: **V106M**, **Y188L**/W, **F227L**, K101A NRTI: M41L, D67N, T69D, V118I, L210W, T215Y, K219R, E44A, V75M, M184T	38 800 (W4)	NNRTI: **V106M**, **F227L**, K101A, Y188W NRTI: M41L, D67N, T69D, V118I, L210W, T215Y, K219R, E44A, V75M, M184T	NNRTI: none NRTI: none	DOR: >max ISL: 7.56
5	P051	B	NNRTI: A98A/G, K103K/N, V106V/I, **Y188**Y/**L** NRTI: M41M/L, E44E/D, D67D/N, K70K/R, V118V/I, M184M/I/V, L210L/W, T215T/N/S/Y, K219K/R	4780 (W12)	NNRTI: A98G, V106I, **Y188L** NRTI: M41L, E44D, V118I, M184V, L210W, T215Y, K219R	NNRTI: none NRTI: none	DOR: >max ISL: 20.00
**Participants with PK results indicating likely nonadherence**							
6	P051	C	NNRTI: none NRTI: none	2220 (W48)	NNRTI: none NRTI: T215T/I	NNRTI: none NRTI: T215T/I	DOR: 0.90 ISL: 1.24
7	P052	B	NNRTI: none NRTI: T69T/N	83 900 (W48)	NNRTI: none NRTI: T69T/N	NNRTI: none NRTI: none	DOR: 0.88 ISL: 1.44

Abbreviations: DOR, doravirine; ISL, islatravir; max, maximum; N/A, not available; NNRTI, non-nucleoside reverse transcriptase inhibitor; NRTI, nucleos(t)ide reverse transcriptase inhibitor; PK, pharmacokinetics; RAM, resistance-associated mutation; W, week.

^a^DOR RAMs are shown in bold.

^b^Resistance testing was attempted on samples from 2 viremia time points in the W36 analysis window.

^c^Participant #3 is not included in the previous analysis tables due to assay failure of baseline resistance testing for all available samples.

^d^Two participants (P051: #4 and #5) that discontinued with confirmed HIV RNA ≥200 copies/mL were later determined to be ineligible for enrollment due to either a previous history of virologic failure (n = 1) or a previous documentation of DOR resistance (n = 1). Both had baseline NNRTI RAMs (including Y188L) and 1 had a baseline M184I/V RAM.

#### Participants Without Postbaseline Results Due to Assay Failure

Three participants (#1, #2, and #3) who met the criteria for postbaseline resistance testing did not have results due to assay failure (Table 7; Supplementary Figure 1). All 3 participants had HIV RNA levels between 200 and 500 copies/mL. Plasma drug levels for these participants were limited but were consistent with study intervention use (Supplementary Table 4).

#### Participants Who Were Later Determined to be Ineligible for Enrollment

Postbaseline genotypic and phenotypic results were available for 4 participants (P051, n = 3; P052, n = 1). Two participants (#4 and #5) from P051 had viremia observed by W4 (Supplementary Figure 2) and had multidrug resistance at baseline and at the last on-treatment viremia visit (including DOR RAMs and changes at M184), with phenotypic fold-shifts appearing as expected based on the genotypic profiles (DOR > max; ISL 7- to 20-fold; Table 7). Both participants had ISL plasma concentrations within the expected range for treatment adherence; DOR plasma concentrations were not evaluated (Supplementary Table 4). Both participants were later determined to be ineligible for enrollment, as previously discussed; these were the only 2 participants with baseline DOR RAMs (including Y188L) that discontinued with viremia (Table 6).

#### Participants With Pharmacokinetic Results Indicating Likely Nonadherence

Two participants (#6 from P051 and #7 from P052) who discontinued at W48 exhibited low or undetectable plasma concentrations of DOR and ISL, indicating likely nonadherence (Supplementary Figure 3 and Supplementary Table 4). Participant #6 had an NRTI revertant, T215T/I, detected at viremia that did not alter the potency of any of the antiretrovirals evaluated (fold-shift 0.55–1.24; Table 7). Participant #7 had T69T/N present at baseline and viremia that did not alter the potency of any of the antiretrovirals evaluated (fold-shift 0.39–1.14).

No participants in the P051 or P052 comparator groups met the criteria for postbaseline resistance analysis through W48.

## DISCUSSION

This analysis evaluated baseline and postbaseline resistance through W48 across three phase 3 studies assessing virologic outcomes following a switch to DOR/ISL (100/0.25 mg) in virologically suppressed adults. As participants were virologically suppressed at study entry, with viremia below the validated limit for viral RNA-based genotypic and phenotypic assays, sequencing of proviral DNA was used to assess baseline resistance. Baseline proviral DNA results were available for >90% of participants and allowed for a thorough analysis of the impact of baseline resistance on viral suppression across 1227 participants treated with DOR/ISL. Among participants with baseline NNRTI RAMs or M184I/V, the levels of viral suppression maintained by DOR/ISL at W48 were similar to those observed across all treated participants in the individual studies and comparable to those of participants without baseline NNRTI RAMs or M184I/V across study arms [24–26]. Viremia outcomes (discontinuations, low-level viremia, and transient viremia) in this population were comparable across treatment groups and studies.

Given the known resistance profiles of DOR and ISL, the baseline resistance analysis focused on NNRTI RAMs and M184I/V, which may reduce DOR or ISL potency, respectively. Results demonstrated comparable viral suppression at W48 across different baseline resistance categories: DOR/ISL maintained HIV RNA <50 copies/mL in 97% of participants with baseline NNRTI RAMs and 94% of participants with baseline M184I/V. Furthermore, the presence or absence of NNRTI RAMs and M184I/V in proviral DNA did not impact viremia outcomes through W48 in participants switching to DOR/ISL (100/0.25 mg). While 7 of 35 participants with baseline NNRTI RAMs and M184I/V experienced viremia, only 3 had not resolved by W48: 1 had low-level viremia and remained in the study, and 2 discontinued prior to W48 (1 was ineligible due to previous documentation of DOR resistance [Y188L] and the other discontinued with HIV RNA <200 copies/mL). Among the 8 participants with DOR RAMs and M184I/V at baseline (Table 6), 6 remained virologically suppressed through W48; 1 participant (with H221H/Y) had transient viremia who resolved by W48, and the other participant (ineligible due to documented Y188L) discontinued. Together, these data indicate that, in the target population, the presence of baseline NNRTI RAMs and M184I/V was not a contributory factor to virologic outcomes.

As the participants in the comparator groups for P051 and P052 were stably suppressed on bART or BIC/FTC/TAF at enrollment and continued their regimen, it was not unexpected that few participants in these groups had HIV RNA ≥50 copies/mL. Additionally, only 30% of participants in the open-label P051 were receiving an NNRTI that could have been affected by the presence of baseline NNRTI RAMs [10, 11].

Of the 4 participants who discontinued treatment and had available postbaseline resistance results after the W48 database lock, no treatment-emergent resistance to either DOR or ISL was detected (Table 7). Two participants had multidrug resistance at baseline (including to DOR) and also M184 mutations in combination with at least 5 thymidine analog mutations in proviral DNA. Both participants were later found to be ineligible for enrollment based on previous medical history or documented DOR RAMs, including Y188L. Postbaseline phenotypic analysis of viral RNA showed complete resistance to DOR and reduced susceptibility to ISL. Across all studies, no other participants who discontinued DOR/ISL treatment due to lack of efficacy had exclusionary DOR RAMs detected at baseline. After W48, post hoc analysis was performed for 1 participant (P052) with baseline NNRTI RAMs (K101K/E, K103K/N) and M184M/V that discontinued at W24 without meeting criteria for postbaseline testing; treatment-emergent DOR resistance was observed with H221Y, M230L, and L234I [23]. The data suggest that in the absence of DOR activity, DOR/ISL was ineffective at maintaining viral suppression. One participant in P051 had T215T/I (a mixture of wild-type and T215I) detected at the last on-treatment viremia with no phenotypic effect observed (all changes in susceptibility across antiretrovirals evaluated were 0.25- to 1.50-fold). T215I is an NRTI RAM classified as a revertant that may indicate prior resistance with T215Y or T215F [27, 28].

While these studies were limited to participants with no history of virologic failure, approximately 20%–25% of participants were detected with baseline NNRTI and NRTI RAMs. As M184I/V appears to decline faster than other RAMs and exhibits a lower replicative capacity, it is unclear whether the observed RAMs are a result of previous experience with these ARVs or from transmitted resistance [29–31]. However, the median time since HIV diagnosis (13.3 years in P051, 11.0 years in P052, and 8.1 years in P054) suggests that many participants may have been exposed to multiple previous regimens [10, 11, 24]. This is supported by data from study P052, in which 35% of participants (119/342) receiving DOR/ISL had previously received >3 ART regimens [11]. One limitation of the analysis is that the studies were not designed to enroll participants with baseline resistance; therefore, the number of participants with mutations associated with DOR and/or ISL was small, precluding a generalizable conclusion. These findings cannot be extrapolated to populations with documented virologic failure.

There has been an increase in the use of proviral DNA as a source for HIV resistance testing, particularly when viral RNA testing is not feasible, as in the current studies, in which virologically suppressed individuals were enrolled [32]. In this study, almost all the RAMs detected at baseline in proviral DNA were also observed postbaseline among participants with available data (Table 7), indicating that RAMs identified in proviral DNA can be useful in predicting what mutations could emerge with loss of virologic suppression. However, variability in the reproducibility of proviral DNA resistance testing has been reported in previous studies; these differences are largely due to inherent sequencing bias rather than assay inaccuracies [33, 34]. While it is possible that low-frequency mutations failed detection, it is possible that mutations that are detected using proviral DNA represent false positives [32, 33]. APOBEC hypermutation can artificially induce RAMs that are not clinically meaningful; however, the GenoSure Archive assay employed for this study includes a proprietary filter and manual quality control at APOBEC-associated sites to reduce APOBEC-induced hypermutation false positivity [19, 35]. Despite this limitation, HIV resistance testing using proviral DNA provides a useful approach, allowing for meaningful assessment of the impact of baseline RAMs on the efficacy of a regimen in the context of a clinical study [33].

## CONCLUSION

Resistance analysis of 1227 virologically suppressed participants who switched to DOR/ISL (100/0.25 mg) in phase 3 studies (P051/P052/P054) indicated that baseline NNRTI RAMs and M184I/V in proviral DNA do not impact the efficacy of DOR/ISL. These results support DOR/ISL as a switch option for people with no history of virologic treatment failure, even in the presence of baseline resistance in proviral DNA.

## Supplementary Material

jiag257_Supplementary_Data

## Supplementary Data

Supplementary materials are available at *The Journal of Infectious Diseases* online (http://jid.oxfordjournals.org/). Supplementary materials consist of data provided by the author that are published to benefit the reader. The posted materials are not copyedited. The contents of all supplementary data are the sole responsibility of the authors. Questions or messages regarding errors should be addressed to the author.
