# Supplementary material for: Switching to Daily Doravirine/Islatravir (100/0.25 mg) Maintains Viral Suppression Through Week 48 Despite Baseline Non-Nucleoside Reverse Transcriptase Inhibitor Resistance–Associated Mutations or M184I/V in Proviral DNA
Source: J Infect Dis. 2026 May 29;234(3):e466–76. doi: 10.1093/infdis/jiag257 (PMC13600411; doi:10.1093/infdis/jiag257)
Supplement: Supplementary file 1 [file jiag257_supplementary_data.docx]

# Supplementary Material

**Table S1. Prevalence of NRTI, NNRTI, InSTI, and PI RAMs in Proviral DNA in P051 at Baseline**

|  | **DOR/ISL** | **bART** |
| --- | --- | --- |
|  | **N=322** | **N=166** |
|  | **n (%)** | **n (%)** |
| **Any NRTI RAM** | **59 (18.3)** | **25 (15.1)** |
| M184M/A/I/T/V | 20 (6.2) | 5 (3.0) |
| M184M/V | 15 (4.7) | 3 (1.8) |
| M184V | 3 (0.9) | 0 |
| M184M/A/T/V | 1 (0.3) | 0 |
| M184M/I/V | 1 (0.3) | 1 (0.6) |
| M184M/I | 0 | 1 (0.6) |
| V118V/I/T | 20 (6.2) | 11 (6.6) |
| V118I | 12 (3.7) | 7 (4.2) |
| V118V/I | 8 (2.5) | 3 (1.8) |
| V118I/T | 0 | 1 (0.6) |
| T215T/A/C/D/F/I/L/N/S/Y | 11 (3.4) | 4 (2.4) |
| T215T/N/S/Y | 3 (0.9) | 1 (0.6) |
| T215T/I | 2 (0.6) | 0 |
| T215D | 1 (0.3) | 1 (0.6) |
| T215S | 1 (0.3) | 0 |
| T215T/A/F | 1 (0.3) | 0 |
| T215T/C/N/S/Y | 1 (0.3) | 0 |
| T215T/F/I/S | 1 (0.3) | 0 |
| T215Y | 1 (0.3) | 0 |
| T215L | 0 | 1 (0.6) |
| T215T/A | 0 | 1 (0.6) |
| K70K/E/R | 10 (3.1) | 2 (1.2) |
| K70K/R | 9 (2.8) | 0 |
| K70K/E | 1 (0.3) | 2 (1.2) |
| K219K/E/N/Q/R | 9 (2.8) | 2 (1.2) |
| K219K/R | 3 (0.9) | 0 |
| K219K/N | 2 (0.6) | 0 |
| K219R | 2 (0.6) | 0 |
| K219E | 1 (0.3) | 0 |
| K219K/E | 1 (0.3) | 1 (0.6) |
| K219Q | 0 | 1 (0.6) |
| D67D/N | 6 (1.9) | 2 (1.2) |
| D67D/N | 5 (1.6) | 2 (1.2) |
| D67N | 1 (0.3) | 0 |
| M41M/I/L | 5 (1.6) | 2 (1.2) |
| M41M/L | 3 (0.9) | 0 |
| M41L | 2 (0.6) | 1 (0.6) |
| M41M/I/L | 0 | 1 (0.6) |
| T69T/D/N/S | 4 (1.2) | 1 (0.6) |
| T69D | 2 (0.6) | 0 |
| T69N/S | 1 (0.3) | 0 |
| T69T/N | 1 (0.3) | 1 (0.6) |
| L210L/W | 3 (0.9) | 0 |
| L210W | 2 (0.6) | 0 |
| L210L/W | 1 (0.3) | 0 |
| E44E/D | 2 (0.6) | 3 (1.8) |
| A62A/V | 1 (0.3) | 1 (0.6) |
| A62V | 1 (0.3) | 0 |
| A62A/V | 0 | 1 (0.6) |
| Q151Q/K/L/M | 1 (0.3) | 0 |
| V75V/I | 1 (0.3) | 1 (0.6) |
| **Any NNRTI RAM** | **79 (24.5)** | **33 (19.9)** |
| K103K/N/R/S | 34 (10.6) | 11 (6.6) |
| K103K/N | 18 (5.6) | 3 (1.8) |
| K103R | 6 (1.9) | 3 (1.8) |
| K103N | 5 (1.6) | 2 (1.2) |
| K103N/S | 3 (0.9) | 0 |
| K103K/N/R/S | 1 (0.3) | 0 |
| K103R/S | 1 (0.3) | 0 |
| K103K/R | 0 | 3 (1.8) |
| E138E/A/D/G/K/Q/T | 21 (6.5) | 7 (4.2) |
| E138A | 11 (3.4) | 4 (2.4) |
| E138E/A | 5 (1.6) | 2 (1.2) |
| E138E/K | 2 (0.6) | 0 |
| E138E/A/K/T | 1 (0.3) | 0 |
| E138E/G | 1 (0.3) | 0 |
| E138K/Q | 1 (0.3) | 0 |
| E138E/A/D | 0 | 1 (0.6) |
| V106V/A/I/M | 13 (4.0) | 4 (2.4) |
| V106V/I | 7 (2.2) | 2 (1.2) |
| V106I | 2 (0.6) | 2 (1.2) |
| V106V/M | 2 (0.6) | 0 |
| V106M | 1 (0.3) | 0 |
| V106V/A | 1 (0.3) | 0 |
| V90V/I | 8 (2.5) | 1 (0.6) |
| V90V/I | 5 (1.6) | 1 (0.6) |
| V90I | 3 (0.9) | 0 |
| V179V/A/D/I/T | 6 (1.9) | 6 (3.6) |
| V179D | 3 (0.9) | 5 (3.0) |
| V179T | 1 (0.3) | 0 |
| V179V/A/I/T | 1 (0.3) | 0 |
| V179V/D | 1 (0.3) | 0 |
| V179V/A/D | 0 | 1 (0.6) |
| G190G/A | 4 (1.2) | 0 |
| A98A/G | 3 (0.9) | 1 (0.6) |
| A98A/G | 3 (0.9) | 0 |
| A98G | 0 | 1 (0.6) |
| Y188Y/C/L/W | 3 (0.9) | 0 |
| Y188L/W | 1 (0.3) | 0 |
| Y188Y/C | 1 (0.3) | 0 |
| Y188Y/L | 1 (0.3) | 0 |
| L100L/I | 2 (0.6) | 0 |
| L100I | 1 (0.3) | 0 |
| L100L/I | 1 (0.3) | 0 |
| V108V/I/M | 2 (0.6) | 3 (1.8) |
| V108V/I | 2 (0.6) | 2 (1.2) |
| V108V/I/M | 0 | 1 (0.6) |
| F227F/L/V | 1 (0.3) | 1 (0.6) |
| F227L | 1 (0.3) | 0 |
| F227F/V | 0 | 1 (0.6) |
| P225P/H | 1 (0.3) | 0 |
| Y181Y/C | 1 (0.3) | 1 (0.6) |
| H221H/Y | 0 | 1 (0.6) |
| K101K/E | 0 | 2 (1.2) |
| P236P/L | 0 | 1 (0.6) |
| Y318F | 0 | 1 (0.6) |
| **Any InSTI RAM** | **124 (38.5)** | **68 (41.0)** |
| M50M/I/T | 91 (28.3) | 53 (31.9) |
| M50I | 61 (18.9) | 39 (23.5) |
| M50M/I | 24 (7.5) | 12 (7.2) |
| M50I/T | 3 (0.9) | 1 (0.6) |
| M50M/I/T | 3 (0.9) | 1 (0.6) |
| E157E/K/Q | 13 (4.0) | 9 (5.4) |
| E157Q | 8 (2.5) | 5 (3.0) |
| E157E/K | 3 (0.9) | 4 (2.4) |
| E157E/Q | 2 (0.6) | 0 |
| G163G/K/Q/R | 9 (2.8) | 4 (2.4) |
| G163G/R | 8 (2.5) | 3 (1.8) |
| G163G/K/Q | 1 (0.3) | 0 |
| G163R | 0 | 1 (0.6) |
| T97T/A/S | 8 (2.5) | 3 (1.8) |
| T97T/A | 4 (1.2) | 2 (1.2) |
| T97A | 3 (0.9) | 1 (0.6) |
| T97A/S | 1 (0.3) | 0 |
| L74L/I/M | 7 (2.2) | 5 (3.0) |
| L74I/M | 3 (0.9) | 1 (0.6) |
| L74L/I/M | 2 (0.6) | 3 (1.8) |
| L74L/M | 1 (0.3) | 0 |
| L74M | 1 (0.3) | 1 (0.6) |
| G140G/A/R/S | 6 (1.9) | 4 (2.4) |
| G140G/R | 3 (0.9) | 4 (2.4) |
| G140G/A | 1 (0.3) | 0 |
| G140G/S | 1 (0.3) | 0 |
| G140S | 1 (0.3) | 0 |
| Q148Q/H/R | 3 (0.9) | 0 |
| Q148Q/R | 2 (0.6) | 0 |
| Q148H | 1 (0.3) | 0 |
| S153S/A | 3 (0.9) | 1 (0.6) |
| S153A | 2 (0.6) | 0 |
| S153S/A | 1 (0.3) | 1 (0.6) |
| T66T/A/I | 3 (0.9) | 0 |
| T66T/I | 2 (0.6) | 0 |
| T66T/A | 1 (0.3) | 0 |
| E138E/K | 2 (0.6) | 1 (0.6) |
| E92E/G | 2 (0.6) | 0 |
| R263R/K | 2 (0.6) | 0 |
| Y143Y/C | 2 (0.6) | 0 |
| N155N/H | 1 (0.3) | 0 |
| **Any PI RAM** | **280 (87.0)** | **149 (89.8)** |
| M36M/I/L/T/V | 166 (51.6) | 87 (52.4) |
| M36I | 128 (39.8) | 74 (44.6) |
| M36M/I | 23 (7.1) | 3 (1.8) |
| M36L | 5 (1.6) | 7 (4.2) |
| M36I/L | 2 (0.6) | 0 |
| M36I/T | 2 (0.6) | 0 |
| M36M/V | 2 (0.6) | 0 |
| M36I/V | 1 (0.3) | 2 (1.2) |
| M36M/I/L | 1 (0.3) | 0 |
| M36M/L | 1 (0.3) | 0 |
| M36V | 1 (0.3) | 0 |
| M36M/I/L/V | 0 | 1 (0.6) |
| H69H/K/N/Q/R/T | 123 (38.2) | 72 (43.4) |
| H69K | 116 (36.0) | 70 (42.2) |
| H69R | 3 (0.9) | 0 |
| H69H/K/N/Q | 1 (0.3) | 0 |
| H69K/Q | 1 (0.3) | 1 (0.6) |
| H69K/R | 1 (0.3) | 1 (0.6) |
| H69K/T | 1 (0.3) | 0 |
| L89L/I/M | 109 (33.9) | 68 (41.0) |
| L89M | 89 (27.6) | 52 (31.3) |
| L89L/M | 8 (2.5) | 8 (4.8) |
| L89I/M | 7 (2.2) | 2 (1.2) |
| L89I | 2 (0.6) | 4 (2.4) |
| L89L/I/M | 2 (0.6) | 2 (1.2) |
| L89L/I | 1 (0.3) | 0 |
| I62I/V | 89 (27.6) | 45 (27.1) |
| I62V | 56 (17.4) | 29 (17.5) |
| I62I/V | 33 (10.2) | 16 (9.6) |
| V77V/I/L/M | 86 (26.7) | 44 (26.5) |
| V77I | 66 (20.5) | 31 (18.7) |
| V77V/I | 18 (5.6) | 13 (7.8) |
| V77I/L | 1 (0.3) | 0 |
| V77V/I/M | 1 (0.3) | 0 |
| K20K/I/M/R/T/V | 48 (14.9) | 22 (13.3) |
| K20R | 28 (8.7) | 11 (6.6) |
| K20K/R | 18 (5.6) | 8 (4.8) |
| K20K/I/T/V | 1 (0.3) | 0 |
| K20M | 1 (0.3) | 0 |
| K20I/M | 0 | 1 (0.6) |
| K20K/R/V | 0 | 1 (0.6) |
| K20K/T | 0 | 1 (0.6) |
| A71A/I/T/V | 43 (13.4) | 18 (10.8) |
| A71T | 13 (4.0) | 5 (3.0) |
| A71A/T | 11 (3.4) | 2 (1.2) |
| A71V | 11 (3.4) | 9 (5.4) |
| A71A/V | 7 (2.2) | 1 (0.6) |
| A71A/I/T/V | 1 (0.3) | 1 (0.6) |
| L10L/F/I/M/V | 42 (13.0) | 25 (15.1) |
| L10I | 14 (4.3) | 7 (4.2) |
| L10L/I | 9 (2.8) | 4 (2.4) |
| L10V | 9 (2.8) | 8 (4.8) |
| L10L/V | 4 (1.2) | 1 (0.6) |
| L10F | 1 (0.3) | 0 |
| L10F/V | 1 (0.3) | 0 |
| L10I/V | 1 (0.3) | 3 (1.8) |
| L10L/F | 1 (0.3) | 0 |
| L10L/F/I | 1 (0.3) | 0 |
| L10L/I/V | 1 (0.3) | 1 (0.6) |
| L10I/M | 0 | 1 (0.6) |
| M46M/I/L/V | 9 (2.8) | 3 (1.8) |
| M46M/I | 4 (1.2) | 1 (0.6) |
| M46M/I/L | 2 (0.6) | 0 |
| M46I | 1 (0.3) | 1 (0.6) |
| M46L | 1 (0.3) | 0 |
| M46M/I/V | 1 (0.3) | 0 |
| M46M/L | 0 | 1 (0.6) |
| I54I/M/T/V | 6 (1.9) | 2 (1.2) |
| I54I/V | 4 (1.2) | 1 (0.6) |
| I54I/M | 1 (0.3) | 0 |
| I54V | 1 (0.3) | 0 |
| I54T | 0 | 1 (0.6) |
| V11V/I | 6 (1.9) | 1 (0.6) |
| V82V/A/L/T | 6 (1.9) | 1 (0.6) |
| V82V/A | 4 (1.2) | 0 |
| V82A/T | 1 (0.3) | 0 |
| V82V/L | 1 (0.3) | 0 |
| V82A | 0 | 1 (0.6) |
| I84I/V | 5 (1.6) | 0 |
| I84I/V | 4 (1.2) | 0 |
| I84V | 1 (0.3) | 0 |
| D30D/N | 4 (1.2) | 4 (2.4) |
| D30D/N | 3 (0.9) | 4 (2.4) |
| D30N | 1 (0.3) | 0 |
| L33L/F | 4 (1.2) | 2 (1.2) |
| L33L/F | 3 (0.9) | 0 |
| L33F | 1 (0.3) | 2 (1.2) |
| L90L/M | 4 (1.2) | 1 (0.6) |
| L90L/M | 4 (1.2) | 0 |
| L90M | 0 | 1 (0.6) |
| G73G/S | 3 (0.9) | 2 (1.2) |
| I47I/V | 3 (0.9) | 0 |
| I85I/V | 3 (0.9) | 1 (0.6) |
| I85I/V | 2 (0.6) | 1 (0.6) |
| I85V | 1 (0.3) | 0 |
| K43K/T | 3 (0.9) | 2 (1.2) |
| K43K/T | 2 (0.6) | 1 (0.6) |
| K43T | 1 (0.3) | 1 (0.6) |
| N88N/D | 2 (0.6) | 0 |
| N88D | 1 (0.3) | 0 |
| N88N/D | 1 (0.3) | 0 |
| I50I/V | 1 (0.3) | 0 |
| L24I/M | 1 (0.3) | 0 |
| Q58Q/E | 1 (0.3) | 0 |
| T74P/S | 1 (0.3) | 0 |
| V32V/I | 1 (0.3) | 0 |
| G48V | 0 | 1 (0.6) |
| N83N/D | 0 | 1 (0.6) |
| Based on genotype data from the Monogram Bioscience GenoSure Archive® assay and the following list of RAMs for each class:  NRTI: M41L, E44D, A62V, K65E/N/R, D67N, T69D/N, 69ins, K70E/R, L74V, V75I, F77L, Y115F, F116Y, V118I, Q151M, M184I/V, L210W, T215A/C/D/E/F/G/H/I/L/N/S/V/Y, K219E/N/Q/R.  NNRTI: V90I, A98G, L100I, K101E/H/P, K103N/R/S, V106A/I/M/T, V108I, E138A/G/K/Q/R, V179D/F/L/T, Y181C/I/V, Y188C/H/L, G190A/E/S, H221Y, P225H, F227C/H/I/L/R/V, M230I/L, L234I, P236L, Y318F.  InSTI: M50I, H51Y, T66A/I/K, L74M, E92G/Q, T97A, G118R, F121Y, E138A/K/T, G140A/C/R/S, Y143C/H/R, S147G, Q148H/K/R, S153A/F/Y, N155H/S, E157K/Q, G163K/R, R263K.  PI: L10F/I/R/V, V11I, K20M/R/T, L24I, D30N, V32I, L33F, M36I/L/V, K43T, M46I/L, I47A/V, G48V, I50L/V, F53L/Y, I54A/L/M/S/T/V, Q58E, I62V, H69K/R, A71T/V, G73A/C/S/T, T74P, L76V, V77I, V82A/F/L/M/S/T, N83D, I84A/C/V, I85V, N88D/S, L89I/M/V, L90M.  Abbreviations: bART, baseline antiretroviral therapy; DOR, doravirine; InSTI, integrase strand transfer inhibitor; ISL, islatravir; N, number of participants with proviral DNA data at baseline; n, participants in the category; NNRTI, non-nucleoside reverse transcriptase inhibitor; NRTI, nucleos(t)ide reverse transcriptase inhibitor; PI, protease inhibitor; RAM, resistance-associated mutation.  Percentage = n/N*100%. | | |

**Table S2. Prevalence of NRTI, NNRTI and InSTI RAMs in Proviral DNA in P052 at Baseline**

|  | **DOR/ISL** | **BIC/FTC/TAF** |
| --- | --- | --- |
|  | **N=309** | **N=152** |
|  | **n (%)** | **n (%)** |
| **Any NRTI RAM** | **62 (20.1)** | **32 (21.1)** |
| M184M/I/V | 24 (7.8) | 10 (6.6) |
| M184M/V | 21 (6.8) | 7 (4.6) |
| M184M/I | 2 (0.6) | 2 (1.3) |
| M184I | 1 (0.3) | 0 |
| M184V | 0 | 1 (0.7) |
| V118V/I | 18 (5.8) | 10 (6.6) |
| V118I | 13 (4.2) | 7 (4.6) |
| V118V/I | 5 (1.6) | 3 (2.0) |
| T215T/A/C/D/E/I/L/N/S/Y | 15 (4.9) | 4 (2.6) |
| T215E | 3 (1.0) | 0 |
| T215T/N/S/Y | 3 (1.0) | 1 (0.7) |
| T215T/I | 2 (0.6) | 0 |
| T215C | 1 (0.3) | 0 |
| T215D | 1 (0.3) | 1 (0.7) |
| T215I/L | 1 (0.3) | 0 |
| T215T/A | 1 (0.3) | 0 |
| T215T/C/D/Y | 1 (0.3) | 0 |
| T215T/C/N/S/Y | 1 (0.3) | 0 |
| T215T/N | 1 (0.3) | 0 |
| T215A | 0 | 1 (0.7) |
| T215T/A/D/N/S/Y | 0 | 1 (0.7) |
| M41M/L | 9 (2.9) | 4 (2.6) |
| M41M/L | 7 (2.3) | 4 (2.6) |
| M41L | 2 (0.6) | 0 |
| K70K/R | 8 (2.6) | 2 (1.3) |
| K70K/R | 6 (1.9) | 2 (1.3) |
| K70R | 2 (0.6) | 0 |
| D67D/N | 7 (2.3) | 3 (2.0) |
| D67D/N | 4 (1.3) | 3 (2.0) |
| D67N | 3 (1.0) | 0 |
| K219K/E/N/Q/R | 6 (1.9) | 4 (2.6) |
| K219K/E | 2 (0.6) | 0 |
| K219E | 1 (0.3) | 0 |
| K219K/N | 1 (0.3) | 1 (0.7) |
| K219K/Q | 1 (0.3) | 1 (0.7) |
| K219K/R | 1 (0.3) | 2 (1.3) |
| T69T/A/D/N | 6 (1.9) | 2 (1.3) |
| T69N | 2 (0.6) | 1 (0.7) |
| T69A/D | 1 (0.3) | 0 |
| T69D | 1 (0.3) | 0 |
| T69T/A/D/N | 1 (0.3) | 1 (0.7) |
| T69T/N | 1 (0.3) | 0 |
| K65K/E/N/R | 4 (1.3) | 1 (0.7) |
| K65K/E | 2 (0.6) | 0 |
| K65K/R | 2 (0.6) | 0 |
| K65K/N | 0 | 1 (0.7) |
| L210L/W | 3 (1.0) | 2 (1.3) |
| V75V/A/I/T | 3 (1.0) | 2 (1.3) |
| V75I | 1 (0.3) | 0 |
| V75V/A/I/T | 1 (0.3) | 0 |
| V75V/I | 1 (0.3) | 2 (1.3) |
| A62A/V | 2 (0.6) | 1 (0.7) |
| A62A/V | 1 (0.3) | 0 |
| A62V | 1 (0.3) | 1 (0.7) |
| L74L/I/V | 2 (0.6) | 3 (2.0) |
| L74L/I/V | 1 (0.3) | 0 |
| L74L/V | 1 (0.3) | 3 (2.0) |
| F77F/L | 1 (0.3) | 0 |
| Q151Q/K/L/M | 1 (0.3) | 0 |
| Y115Y/F | 0 | 1 (0.7) |
| **Any NNRTI RAM** | **82 (26.5)** | **41 (27.0)** |
| K103K/N/R/S | 38 (12.3) | 16 (10.5) |
| K103K/N | 13 (4.2) | 4 (2.6) |
| K103N | 11 (3.6) | 4 (2.6) |
| K103R | 5 (1.6) | 4 (2.6) |
| K103K/R | 3 (1.0) | 3 (2.0) |
| K103K/N/R/S | 2 (0.6) | 0 |
| K103N/S | 2 (0.6) | 0 |
| K103S | 2 (0.6) | 1 (0.7) |
| E138E/A/G/Q | 18 (5.8) | 9 (5.9) |
| E138A | 10 (3.2) | 6 (3.9) |
| E138E/A | 5 (1.6) | 2 (1.3) |
| E138G | 2 (0.6) | 0 |
| E138Q | 1 (0.3) | 0 |
| E138E/G | 0 | 1 (0.7) |
| G190G/A/E/S | 9 (2.9) | 4 (2.6) |
| G190G/A | 4 (1.3) | 1 (0.7) |
| G190G/E | 2 (0.6) | 1 (0.7) |
| G190G/S | 2 (0.6) | 0 |
| G190A | 1 (0.3) | 2 (1.3) |
| V106V/I/M | 9 (2.9) | 10 (6.6) |
| V106I | 4 (1.3) | 3 (2.0) |
| V106V/I | 4 (1.3) | 7 (4.6) |
| V106V/I/M | 1 (0.3) | 0 |
| V90V/I | 8 (2.6) | 4 (2.6) |
| V90I | 4 (1.3) | 1 (0.7) |
| V90V/I | 4 (1.3) | 3 (2.0) |
| Y181Y/C/V | 7 (2.3) | 1 (0.7) |
| Y181Y/C | 5 (1.6) | 1 (0.7) |
| Y181C | 1 (0.3) | 0 |
| Y181V | 1 (0.3) | 0 |
| K101K/E | 5 (1.6) | 4 (2.6) |
| K101K/E | 3 (1.0) | 3 (2.0) |
| K101E | 2 (0.6) | 1 (0.7) |
| H221H/Y | 4 (1.3) | 1 (0.7) |
| P225P/H | 3 (1.0) | 1 (0.7) |
| V108V/I | 3 (1.0) | 2 (1.3) |
| M230M/L | 2 (0.6) | 0 |
| V179V/A/D/E | 2 (0.6) | 4 (2.6) |
| V179D/E | 1 (0.3) | 0 |
| V179V/D | 1 (0.3) | 2 (1.3) |
| V179D | 0 | 1 (0.7) |
| V179V/A/D | 0 | 1 (0.7) |
| Y318Y/F | 2 (0.6) | 0 |
| A98A/G | 1 (0.3) | 1 (0.7) |
| A98G | 1 (0.3) | 0 |
| A98A/G | 0 | 1 (0.7) |
| L100L/I | 1 (0.3) | 0 |
| L234L/I | 1 (0.3) | 0 |
| Y188Y/C/F/H/L | 1 (0.3) | 1 (0.7) |
| Y188Y/C | 1 (0.3) | 0 |
| Y188Y/F/H/L | 0 | 1 (0.7) |
| **Any InSTI RAM** | **88 (28.5)** | **37 (24.3)** |
| M50M/I/T | 49 (15.9) | 23 (15.1) |
| M50I | 34 (11.0) | 17 (11.2) |
| M50M/I | 13 (4.2) | 6 (3.9) |
| M50M/I/T | 2 (0.6) | 0 |
| E157E/K/Q | 19 (6.1) | 9 (5.9) |
| E157Q | 14 (4.5) | 4 (2.6) |
| E157E/K | 4 (1.3) | 4 (2.6) |
| E157E/Q | 1 (0.3) | 1 (0.7) |
| T97T/A | 8 (2.6) | 0 |
| T97T/A | 5 (1.6) | 0 |
| T97A | 3 (1.0) | 0 |
| L74L/I/M | 7 (2.3) | 1 (0.7) |
| L74L/I/M | 3 (1.0) | 0 |
| L74L/M | 2 (0.6) | 0 |
| L74M | 2 (0.6) | 0 |
| L74I/M | 0 | 1 (0.7) |
| E138E/K | 3 (1.0) | 0 |
| E138E/K | 2 (0.6) | 0 |
| E138K | 1 (0.3) | 0 |
| G163G/K/R | 3 (1.0) | 2 (1.3) |
| G163G/R | 2 (0.6) | 2 (1.3) |
| G163G/K | 1 (0.3) | 0 |
| S147S/G | 2 (0.6) | 0 |
| T66T/I | 2 (0.6) | 0 |
| E92E/G | 1 (0.3) | 1 (0.7) |
| G140G/R | 1 (0.3) | 0 |
| N155N/S | 1 (0.3) | 2 (1.3) |
| R263R/K | 1 (0.3) | 0 |
| S153S/A | 1 (0.3) | 0 |
| Based on genotype data from the Monogram Bioscience GenoSure Archive® assay and the following list of RAMs:  NRTI: M41L, E44D, A62V, K65E/N/R, D67N, T69D/N, 69ins, K70E/R, L74V, V75I, F77L, Y115F, F116Y, V118I, Q151M, M184I/V, L210W, T215A/C/D/E/F/G/H/I/L/N/S/V/Y, K219E/N/Q/R.  NNRTI: V90I, A98G, L100I, K101E/H/P, K103N/R/S, V106A/I/M/T, V108I, E138A/G/K/Q/R, V179D/F/L/T, Y181C/I/V, Y188C/H/L, G190A/E/S, H221Y, P225H, F227C/H/I/L/R/V, M230I/L, L234I, P236L, Y318F.  InSTI: M50I, H51Y, T66A/I/K, L74M, E92G/Q, T97A, G118R, F121Y, E138A/K/T, G140A/C/R/S, Y143C/H/R, S147G, Q148H/K/R, S153A/F/Y, N155H/S, E157K/Q, G163K/R, R263K.  Abbreviations: BIC, bictegravir; DOR, doravirine; FTC, emtricitabine; InSTI, integrase strand transfer inhibitor; ISL, islatravir; N, number of participants with proviral DNA data at baseline; n, participants in the category; NNRTI, non-nucleoside reverse transcriptase inhibitor; NRTI, nucleos(t)ide reverse transcriptase inhibitor; RAM, resistance-associated mutation; TAF, tenofovir alafenamide.  Percentage = n/N*100%. | | |

**Table S3. Prevalence of NRTI and NNRTI RAMs in Proviral DNA in P054 at Baseline**

|  | DOR/ISL |
| --- | --- |
|  | N=596 |
|  | n (%) |
| **Any NRTI RAMs** | **132 (22.1)** |
| V118V/I | 49 (8.2) |
| V118I | 37 (6.2) |
| V118V/I | 12 (2.0) |
| M184M/I/V | 26 (4.4) |
| M184M/V | 18 (3.0) |
| M184M/I | 4 (0.7) |
| M184M/I/V | 2 (0.3) |
| M184V | 2 (0.3) |
| T215T/C/D/E/F/I/L/N/S/Y | 21 (3.5) |
| T215S | 5 (0.8) |
| T215T/F/I/S | 3 (0.5) |
| T215C | 2 (0.3) |
| T215D | 2 (0.3) |
| T215T/I | 2 (0.3) |
| T215Y | 2 (0.3) |
| T215C/S | 1 (0.2) |
| T215L | 1 (0.2) |
| T215T/E | 1 (0.2) |
| T215T/F/I/N/S/Y | 1 (0.2) |
| T215T/N/S/Y | 1 (0.2) |
| A62A/V | 17 (2.9) |
| A62V | 13 (2.2) |
| A62A/V | 4 (0.7) |
| K219K/E/N/Q/R | 14 (2.3) |
| K219K/Q | 4 (0.7) |
| K219K/R | 4 (0.7) |
| K219K/E | 2 (0.3) |
| K219E | 1 (0.2) |
| K219K/N | 1 (0.2) |
| K219N | 1 (0.2) |
| K219Q | 1 (0.2) |
| K70K/E/G/R | 14 (2.3) |
| K70K/R | 8 (1.3) |
| K70K/E | 3 (0.5) |
| K70R | 2 (0.3) |
| K70K/E/G/R | 1 (0.2) |
| M41M/I/L | 11 (1.8) |
| M41L | 7 (1.2) |
| M41M/L | 3 (0.5) |
| M41M/I/L | 1 (0.2) |
| D67D/N | 9 (1.5) |
| D67D/N | 6 (1.0) |
| D67N | 3 (0.5) |
| T69T/A/D/N | 9 (1.5) |
| T69T/N | 4 (0.7) |
| T69N | 3 (0.5) |
| T69D | 1 (0.2) |
| T69T/A/D/N | 1 (0.2) |
| K65K/E/R | 3 (0.5) |
| K65K/R | 2 (0.3) |
| K65K/E | 1 (0.2) |
| L210L/W | 3 (0.5) |
| L210L/W | 2 (0.3) |
| L210W | 1 (0.2) |
| V75V/I/T | 3 (0.5) |
| V75I | 1 (0.2) |
| V75I/T | 1 (0.2) |
| V75V/I | 1 (0.2) |
| E44E/D | 2 (0.3) |
| E44D | 1 (0.2) |
| E44E/D | 1 (0.2) |
| L74V | 1 (0.2) |
| Y115Y/F | 1 (0.2) |
| **Any NNRTI RAMs** | **162 (27.2)** |
| K103K/H/N/R/S/T | 53 (8.9) |
| K103N | 15 (2.5) |
| K103R | 14 (2.3) |
| K103K/R | 10 (1.7) |
| K103K/N | 9 (1.5) |
| K103K/N/R/S | 2 (0.3) |
| K103H/N | 1 (0.2) |
| K103R/T | 1 (0.2) |
| K103S | 1 (0.2) |
| E138E/A/G/K/S | 39 (6.5) |
| E138A | 21 (3.5) |
| E138E/A | 9 (1.5) |
| E138E/K | 4 (0.7) |
| E138E/G | 2 (0.3) |
| E138K | 2 (0.3) |
| E138A/S | 1 (0.2) |
| V179V/A/D/E/I/L/N/T | 26 (4.4) |
| V179V/D | 9 (1.5) |
| V179D | 8 (1.3) |
| V179T | 3 (0.5) |
| V179D/E | 2 (0.3) |
| V179A/D | 1 (0.2) |
| V179I/L | 1 (0.2) |
| V179V/A/I/T | 1 (0.2) |
| V179V/D/I/N | 1 (0.2) |
| V106V/A/I/M | 25 (4.2) |
| V106V/I | 11 (1.8) |
| V106I | 10 (1.7) |
| V106V/A | 2 (0.3) |
| V106V/I/M | 1 (0.2) |
| V106V/M | 1 (0.2) |
| V90V/I | 23 (3.9) |
| V90V/I | 12 (2.0) |
| V90I | 11 (1.8) |
| K101K/A/E/P/T | 11 (1.8) |
| K101K/E | 7 (1.2) |
| K101E | 2 (0.3) |
| K101K/A/E/T | 1 (0.2) |
| K101P | 1 (0.2) |
| G190G/A/E/Q/S | 10 (1.7) |
| G190A | 3 (0.5) |
| G190G/A | 2 (0.3) |
| G190E | 1 (0.2) |
| G190G/A/S | 1 (0.2) |
| G190G/E | 1 (0.2) |
| G190G/E/Q | 1 (0.2) |
| G190G/S | 1 (0.2) |
| V108V/I | 7 (1.2) |
| H221H/Y | 3 (0.5) |
| P225P/H | 3 (0.5) |
| Y181Y/C | 3 (0.5) |
| Y181Y/C | 2 (0.3) |
| Y181C | 1 (0.2) |
| F227F/C/L | 2 (0.3) |
| F227F/C | 1 (0.2) |
| F227F/L | 1 (0.2) |
| M230M/I/L | 2 (0.3) |
| M230M/I | 1 (0.2) |
| M230M/L | 1 (0.2) |
| Y188Y/H | 2 (0.3) |
| A98G | 1 (0.2) |
| Based on genotype data from the Monogram Bioscience GenoSure Archive® assay and the following list of RAMs:  NRTI: M41L, E44D, A62V, K65E/N/R, D67N, T69D/N, 69ins, K70E/R, L74V, V75I, F77L, Y115F, F116Y, V118I, Q151M, M184I/V, L210W, T215A/C/D/E/F/G/H/I/L/N/S/V/Y, K219E/N/Q/R.  NNRTI: V90I, A98G, L100I, K101E/H/P, K103N/R/S, V106A/I/M/T, V108I, E138A/G/K/Q/R, V179D/F/L/T, Y181C/I/V, Y188C/H/L, G190A/E/S, H221Y, P225H, F227C/H/I/L/R/V, M230I/L, L234I, P236L, Y318F.  Abbreviations: DOR, doravirine; ISL, islatravir; N, number of participants with proviral DNA data at baseline; n, participants in the category; NNRTI, non-nucleoside reverse transcriptase inhibitor; NRTI, nucleos(t)ide reverse transcriptase inhibitor; RAM, resistance-associated mutation.  Percentage = n/N*100%. | |

**Table S4. Adherence Assessment Based on DOR and ISL Plasma Concentrations for Participants With Post-Baseline Analysis**

| **Participant** | **Study** | **Study Visit** | **Day** | **Time Since Last Dose (h)** | **Plasma DOR (nM)^a^** | **Plasma ISL (nM)^a^** | **Adherence Assessment (Y/N)^b^** | |
| --- | --- | --- | --- | --- | --- | --- | --- | --- |
| **1** | P052 | Week 4 | 29 | 24.6 | 981 | 0.582 | Y | |
|  |  | Week 4 | 29 | 0.6 | NA | 9.79 | Y | |
|  |  | Week 24 | 191 | 0.7 | 1280 | 0.609 | Y | |
|  |  | Week 36 | 247 | 0.5 | 1180 | 0.594 | Y | |
|  |  | Week 36 | 275 | 0.2 | 1380 | 0.308 | Y | |
|  |  | Discontinuation visit | 299 | 99.2 | 20.6 | 0.362 | Y | |
| **2** | P054 | Week 4 | 29 | 18.9 | NA | 1.02 | Y | |
|  |  | Week 12 | 113 | 19.1 | NA | 0.984 | Y | |
|  |  | Discontinuation visit | 127 | 18.8 | NA | 1.01 | Y | |
| **3** | P054 | Week 4 | 29 | 23.2 | NA | 1.22 | Y | |
|  |  | Week 48 | 371 | 23.4 | NA | 1.2 | Y | |
|  |  | Discontinuation visit | 400 | 23.3 | NA | 0.978 | Y | |
| **4** | P051 | Week 4 | 27 | 4 | NA | 2.9 | Y | |
|  |  | Week 4 | 27 | 4 | NA | 2.77 | Y | |
|  |  | Week 4 | 31 | 5.7 | NA | 2.11 | Y | |
|  |  | Week 4 | 31 | 5.7 | NA | 1.91 | Y | |
|  |  | Discontinuation visit | 38 | 29 | NA | 0.885 | Y | |
| **5** | P051 | Week 4 | 27 | 32.1 | NA | 0.588 | Y | |
|  |  | Week 4 | 27 | 0.6 | NA | 6.65 | Y | |
|  |  | Week 4 | 57 | 23.4 | NA | 0.698 | Y | |
|  |  | Discontinuation visit | 72 | 1.7 | NA | 7.02 | Y | |
| **6** | P051 | Week 4 | 35 | 13.7 | BLQ | 0.287 | N | |
|  |  | Week 12 | 92 | 13 | 947 | 0.376 | Y | |
|  |  | Week 24 | 165 | 14 | 256 | 0.294 | Y | |
|  |  | Week 36 | 246 | 15.1 | BLQ | BLQ | N | |
|  |  | Week 36 | 292 | 15.6 | BLQ | BLQ | N | |
|  |  | Week 48 | 329 | 0.8 | BLQ | BLQ | N | |
|  |  | Week 48 | 354 | 433.7 | BLQ | BLQ | N | |
| **7** | P052 | Week 4 | 29 | 17 | 1390 | 0.412 | Y | |
|  |  | Week 12 | 79 | 17 | 1740 | 0.514 | Y | |
|  |  | Week 24 | 169 | 14.7 | NA | 0.55 | Y | |
|  |  | Week 36 | 253 | 15.6 | 9.91 | BLQ | N | |
|  |  | Week 48 | 331 | 21.3 | 2.42 | BLQ | N | |
|  |  | Discontinuation visit | 346 | 15.7 | BLQ | BLQ | N | |
| Abbreviations: BLQ, below the limit of quantification; DOR, doravirine; ISL, islatravir; LLOQ, lower limit of quantification; N, no; NA, not available; Y, yes.  ^a^LLOQ for DOR = 0.068 nM and for ISL = 2.35 nM.  ^b^Adherence assessment is shown for each visit as Y or N considering whether the DOR and/or ISL levels are within the expected ranges based upon the time since last dose provided by the participant at the study visit. | | | | | | | |  |

**Figure S1. Viremia over time for DOR/ISL participants #1 and #2 without post-baseline resistance results due to assay failure.**

Initial and reflex values (when available) are displayed in order of assay performed. HIV-1 RNA values are shown as measured. For presentation purposes, values with no HIV-1 RNA detected are displayed as 0 copies/mL and values with <20 copies/mL are displayed as 10 copies/mL. Vertical dashed line indicates the time of discontinuation from study intervention. Points with open circles were obtained after discontinuation of study intervention. Time points with post-baseline resistance testing attempted but resulting in assay failure are circled in each graph.

c, copies.

**Figure S2. Viremia over time for DOR/ISL participants #4 and #5 who were later determined to be ineligible for enrollment.**

Initial and reflex values (when available) are displayed in order of assay performed. HIV-1 RNA values are shown as measured. For presentation purposes, values with no HIV-1 RNA detected are displayed as 0 copies/mL and values with <20 copies/mL are displayed as 10 copies/mL. Vertical dashed line indicates the time of discontinuation. Points with open circles were obtained from after discontinuation of study intervention. Samples assessed with post-baseline resistance results displayed in Table 7 are circled in each graph.

c, copies

**Figure S3. Viremia over time for DOR/ISL participants #6 and #7 who had PK results indicating likely nonadherence.**

Initial and reflex values (when available) are displayed in order of assay performed. HIV-1 RNA values are shown as measured. For presentation purposes, values with no HIV-1 RNA detected are displayed as 0 copies/mL and values with <20 copies/mL are displayed as 10 copies/mL. Horizontal dashed lines are included for 200 copies/mL and 50 copies/mL. Vertical dashed line indicates the time of discontinuation. Samples assessed with post-baseline resistance results displayed in Table 7 are circled in each graph.

c, copies
